# Supplementary material for: Bayesian refinement of protein structures and ensembles against SAXS data using molecular dynamics
Source: PLoS Comput Biol. 2017 Oct 18;13(10):e1005800. doi: 10.1371/journal.pcbi.1005800 (PMC5662244; doi:10.1371/journal.pcbi.1005800)
Supplement: S1 Archive — (BZ2) [file pcbi.1005800.s011.tar.bz2 › gromacs_4.62_bayesian_saxs_refi/share/html/online/g_hbond.html]

g\_hbond


| g\_hbond Main Table of Contents |

**VERSION 4.6  
Sat 19 Jan 2013**

---

### Description

g\_hbond computes and analyzes hydrogen bonds. Hydrogen bonds are
determined based on cutoffs for the angle Hydrogen - Donor - Acceptor
(zero is extended) and the distance Donor - Acceptor
(or Hydrogen - Acceptor using -noda).
OH and NH groups are regarded as donors, O is an acceptor always,
N is an acceptor by default, but this can be switched using
-nitacc. Dummy hydrogen atoms are assumed to be connected
to the first preceding non-hydrogen atom.

You need to specify two groups for analysis, which must be either
identical or non-overlapping. All hydrogen bonds between the two
groups are analyzed.

If you set -shell, you will be asked for an additional index group
which should contain exactly one atom. In this case, only hydrogen
bonds between atoms within the shell distance from the one atom are
considered.

With option -ac, rate constants for hydrogen bonding can be derived with the model of Luzar and Chandler
(Nature 394, 1996; J. Chem. Phys. 113:23, 2000) or that of Markovitz and Agmon (J. Chem. Phys 129, 2008).
If contact kinetics are analyzed by using the -contact option, then
n(t) can be defined as either all pairs that are not within contact distance r at time t
(corresponding to leaving the -r2 option at the default value 0) or all pairs that
are within distance r2 (corresponding to setting a second cut-off value with option -r2).
See mentioned literature for more details and definitions.

[ selected ]  
20 21 24  
25 26 29  
1 3 6  
  
Note that the triplets need not be on separate lines.
Each atom triplet specifies a hydrogen bond to be analyzed,
note also that no check is made for the types of atoms.

**Output:**  
-num: number of hydrogen bonds as a function of time.  
-ac: average over all autocorrelations of the existence
functions (either 0 or 1) of all hydrogen bonds.  
-dist: distance distribution of all hydrogen bonds.  
-ang: angle distribution of all hydrogen bonds.  
-hx: the number of n-n+i hydrogen bonds as a function of time
where n and n+i stand for residue numbers and i ranges from 0 to 6.
This includes the n-n+3, n-n+4 and n-n+5 hydrogen bonds associated
with helices in proteins.  
-hbn: all selected groups, donors, hydrogens and acceptors
for selected groups, all hydrogen bonded atoms from all groups and
all solvent atoms involved in insertion.  
-hbm: existence matrix for all hydrogen bonds over all
frames, this also contains information on solvent insertion
into hydrogen bonds. Ordering is identical to that in -hbn
index file.  
-dan: write out the number of donors and acceptors analyzed for
each timeframe. This is especially useful when using -shell.  
-nhbdist: compute the number of HBonds per hydrogen in order to
compare results to Raman Spectroscopy.

Note: options -ac, -life, -hbn and -hbm
require an amount of memory proportional to the total numbers of donors
times the total number of acceptors in the selected group(s).

### Files

| option | filename | type | description |
| --- | --- | --- | --- |
| **-f** | traj.xtc | Input | Trajectory: xtc trr trj gro g96 pdb cpt |
| **-s** | topol.tpr | Input | Run input file: tpr tpb tpa |
| **-n** | index.ndx | Input, Opt. | Index file |
| **-num** | hbnum.xvg | Output | xvgr/xmgr file |
| **-g** | hbond.log | Output, Opt. | Log file |
| **-ac** | hbac.xvg | Output, Opt. | xvgr/xmgr file |
| **-dist** | hbdist.xvg | Output, Opt. | xvgr/xmgr file |
| **-ang** | hbang.xvg | Output, Opt. | xvgr/xmgr file |
| **-hx** | hbhelix.xvg | Output, Opt. | xvgr/xmgr file |
| **-hbn** | hbond.ndx | Output, Opt. | Index file |
| **-hbm** | hbmap.xpm | Output, Opt. | X PixMap compatible matrix file |
| **-don** | donor.xvg | Output, Opt. | xvgr/xmgr file |
| **-dan** | danum.xvg | Output, Opt. | xvgr/xmgr file |
| **-life** | hblife.xvg | Output, Opt. | xvgr/xmgr file |
| **-nhbdist** | nhbdist.xvg | Output, Opt. | xvgr/xmgr file |

### Other options

| option | type | default | description |
| --- | --- | --- | --- |
| **-[no]h** | bool | no | Print help info and quit || **-[no]version** | bool | no | Print version info and quit || **-nice** | int | 19 | Set the nicelevel || **-b** | time | 0 | First frame (ps) to read from trajectory || **-e** | time | 0 | Last frame (ps) to read from trajectory || **-dt** | time | 0 | Only use frame when t MOD dt = first time (ps) || **-tu** | enum | ps | Time unit: fs, ps, ns, us, ms or s || **-xvg** | enum | xmgrace | xvg plot formatting: xmgrace, xmgr or none || **-a** | real | 30 | Cutoff angle (degrees, Hydrogen - Donor - Acceptor) || **-r** | real | 0.35 | Cutoff radius (nm, X - Acceptor, see next option) || **-[no]da** | bool | yes | Use distance Donor-Acceptor (if TRUE) or Hydrogen-Acceptor (FALSE) || **-r2** | real | 0 | Second cutoff radius. Mainly useful with -contact and -ac || **-abin** | real | 1 | Binwidth angle distribution (degrees) || **-rbin** | real | 0.005 | Binwidth distance distribution (nm) || **-[no]nitacc** | bool | yes | Regard nitrogen atoms as acceptors || **-[no]contact** | bool | no | Do not look for hydrogen bonds, but merely for contacts within the cut-off distance || **-shell** | real | -1 | when > 0, only calculate hydrogen bonds within # nm shell around one particle || **-fitstart** | real | 1 | Time (ps) from which to start fitting the correlation functions in order to obtain the forward and backward rate constants for HB breaking and formation. With -gemfit we suggest -fitstart 0 || **-fitstart** | real | 1 | Time (ps) to which to stop fitting the correlation functions in order to obtain the forward and backward rate constants for HB breaking and formation (only with -gemfit) || **-temp** | real | 298.15 | Temperature (K) for computing the Gibbs energy corresponding to HB breaking and reforming || **-smooth** | real | -1 | If >= 0, the tail of the ACF will be smoothed by fitting it to an exponential function: y = A exp(-x/τ) || **-dump** | int | 0 | Dump the first N hydrogen bond ACFs in a single .xvg file for debugging || **-max\_hb** | real | 0 | Theoretical maximum number of hydrogen bonds used for normalizing HB autocorrelation function. Can be useful in case the program estimates it wrongly || **-[no]merge** | bool | yes | H-bonds between the same donor and acceptor, but with different hydrogen are treated as a single H-bond. Mainly important for the ACF. || **-geminate** | enum | none | Use reversible geminate recombination for the kinetics/thermodynamics calclations. See Markovitch et al., J. Chem. Phys 129, 084505 (2008) for details.: none, dd, ad, aa or a4 || **-diff** | real | -1 | Dffusion coefficient to use in the reversible geminate recombination kinetic model. If negative, then it will be fitted to the ACF along with ka and kd. || **-nthreads** | int | 0 | Number of threads used for the parallel loop over autocorrelations. nThreads <= 0 means maximum number of threads. Requires linking with OpenMP. The number of threads is limited by the number of processors (before OpenMP v.3 ) or environment variable OMP\_THREAD\_LIMIT (OpenMP v.3) || **-acflen** | int | -1 | Length of the ACF, default is half the number of frames || **-[no]normalize** | bool | yes | Normalize ACF || **-P** | enum | 0 | Order of Legendre polynomial for ACF (0 indicates none): 0, 1, 2 or 3 || **-fitfn** | enum | none | Fit function: none, exp, aexp, exp\_exp, vac, exp5, exp7, exp9 or erffit || **-ncskip** | int | 0 | Skip this many points in the output file of correlation functions || **-beginfit** | real | 0 | Time where to begin the exponential fit of the correlation function || **-endfit** | real | -1 | Time where to end the exponential fit of the correlation function, -1 is until the end |

### Known problems

- The option -sel that used to work on selected hbonds is out of order, and therefore not available for the time being.

---

http://www.gromacs.org  
gromacs@gromacs.org
